# Patient Goals and Motivation for Thumb Carpometacarpal Osteoarthritis Surgery

**DOI:** 10.1177/1558944720940063

**Published:** 2020-09-28

**Authors:** Else Marit Holen Gravås, Ingvild Kjeken, Randi Nossum, Ruth Else Mehl Eide, Åse Klokkeide, Karin Hoegh Matre, Monika Olsen, Øyvor Andreassen, Ida K. Haugen, Nina Østerås, Anne Therese Tveter

**Affiliations:** 1 Diakonhjemmet Hospital, Oslo, Norway; 2 Oslo Metropolitan University, Norway; 3 St. Olavs Hospital, Trondheim University Hospital, Norway; 4 Haukeland University Hospital, Bergen, Norway; 5 Haugesund Rheumatism Hospital AS, Norway

**Keywords:** hand, thumb carpometacarpal joint, osteoarthritis, occupational therapy, surgery

## Abstract

**Background:**

Knowledge is lacking on patient goals and motivation for carpometacarpal joint osteoarthritis (CMCJ OA) surgery. The objective of this study was to explore patient goals and motivation for surgery, whether patient goals were reflected in self-reports of pain and function, and factors characterizing patients highly motivated for surgery.

**Methods:**

This cross-sectional study included 180 patients referred from their general practitioner for CMCJ surgical consultation. Goals for surgery were collected with an open-ended question, categorized with the International Classification of Functioning, Disability and Health coding system, and compared to self-reports of pain and function. Motivation for surgery was rated with a Numeric Rating Scale (NRS, 0-10, 0 = not motivated). Factors characterizing patients highly motivated for surgery (NRS ≥ 8) were explored with multivariate regression analyses.

**Results:**

The mean age of the participants was 63 years (SD = 7.6), and 142 (79%) were women. The most common goals for surgery were to reduce pain and improve arm and hand use, but these were not reflected in self-reports of pain and function. Fifty-six (31%) of the patients were characterized as highly motivated for surgery. High motivation for surgery was strongly associated with reporting more activity limitations (odds ratio [OR] = 4.00, *P* = .008), living alone (OR = 3.18, *P* = .007), and a young age (OR = 0.94, *P* = .002).

**Conclusions:**

Decisions on CMCJ OA surgery should be based on assessment and discussion of patients’ life situation, hand pain, activity limitations for, and goals and motivation for surgery. According to the european league against rheumatism (EULAR) recommendations, previously received conservative and pharmacological treatment should also be evaluated.

## Introduction

Osteoarthritis (OA) in the thumb carpometacarpal joint (CMCJ OA) is a prevalent joint disease that can cause severe pain and stiffness and can lead to limitations in activity, restricted participation in work and social life activities, and reduced health-related quality of life.^1,2^ The radiographic prevalence of CMCJ OA is approximately 30% in the adult population.^3^ This prevalence increases with age, and it is higher among women than men.^4^

There is currently no cure or disease-modifying treatment for OA. With the primary aim of reducing pain and improving function, nonpharmacological interventions are recommended as a core treatment for all patients with hand OA.^5,6^ These interventions include patient education on hand OA and ergonomic principles, the use of assistive devices, and an individualized hand exercise program. In addition, patients with CMCJ OA might benefit from thumb orthoses, which can increase CMCJ stability, prevent deformities, and reduce pain.^7-9^ Pharmacological therapy is recommended as a symptom-relieving supplement.^10^ Surgery should only be considered when nonsurgical interventions have yielded unsatisfactory outcomes. The main indications for surgery are persistent pain and reduced function^5,11,12^ in joints with evidence of radiographic OA. Surgical techniques might provide pain relief and improve function, but results vary with the stage and the nature of the disease.^13,14^ No surgical procedure has been shown to be superior to another, and postoperative complications are frequently reported.^12,15^ Sick leave and postoperative rehabilitation are typically required.^16-18^

Shared decision-making between patients and health care professionals is recommended to ensure that treatment choices are based on relevant knowledge of the benefits and risks of different treatment options.^19,20^ However, patient treatment choices can be based on various factors. In a retrospective study of patients with CMCJ OA, participants with less hand, arm, and shoulder disabilities were less likely to choose surgery than those with higher disability.^21^ Similarly, in a prospective study of patients with CMCJ OA, patients that experienced reduced pain during preoperative conservative treatments were less likely to undergo surgery.^6^ These findings are consistent with a study on hip and knee OA, where less pain and higher health-related quality of life were associated with less willingness to consider surgery.^22^ In contrast, Selten et al^23^ showed that symptom severity in hip and knee OA was less important in their choice of treatment than the individual’s positive beliefs about treatment modalities. Furthermore, Traumer et al^24^ found that the interaction between the doctor and the patient with OA was a highly influential factor in the patient’s decision-making process. Recent research has also shown that high motivation for surgery was a significant predictor of the decision to undergo CMCJ surgery.^25^ However, more knowledge is needed to clarify which factors motivate patients with CMCJ OA to undergo surgery. The objective of this study was to explore: (1) patient goals for CMCJ surgery; (2) whether patient goals were reflected in self-reports of pain and function; and (3) factors that characterized patients highly motivated for surgery.

## Material and Methods

### Study Design

This cross-sectional study acquired baseline data from a multicenter randomized controlled trial (RCT), in which the main aim was to examine whether occupational therapy, provided in the time period between referral and surgical consultation, might delay or reduce the need for surgery in patients with CMCJ OA over a 2-year period^26^ (Trial registration: NCT01794754).

### Participants

Participants were patients who based on their general practitioners’ (GP) clinical judgment, were referred to a CMCJ OA surgical consultation at 3 hospitals in Norway: St.Olav’s University Hospital in Trondheim, Haukeland University Hospital in Bergen, and Haugesund Rheumatism Hospital in Haugesund. Study protocol details and prior analyses of these patients have been previously published.^25-27^ Patients with a poor understanding of the language or cognitive dysfunctions were excluded. Written informed consent was collected from all patients prior to the baseline assessment. The Regional Committees for Medical Research Ethics approved the study (Ref. no: 2012/2265/REK South East Norway).

### Measures

At baseline, patients answered an open-ended question about their goal for CMCJ surgery: *“What is the most important goal you want to achieve by undergoing surgery*?” When patients could not formulate a specific goal for surgery, they were given examples; that is, asked whether their goal was related to reducing pain in the thumb, improving activity performance, or increasing grip function or grip strength, because these are the most commonly reported functional consequences of CMCJ OA.^28^ Furthermore, they were asked to rate their motivation for surgery on a Numeric Rating Scale (NRS) of 0-10, where 0 = not motivated at all and 10 = very motivated.

In this study, the International Classification of Functioning, Disability, and Health (ICF) model^29^ was used as a theoretical and analytical framework. The model provides a common understanding of the term “functioning,” which refers to body functions, body structures, activities, and participation, and also includes environmental and personal factors.

Personal factors collected in this study included age, gender, marital status (living alone/living with someone), and work status. Body structures comprised severity of radiographic CMCJ OA and was classified using the Modified Kellgren-Lawrence grade (KLG) scale (Grades 0-4: 0 = no CMCJ OA, 4 = severe OA).^3^ Patients with a KLG-scores ≥ 2 were categorized as having CMCJ OA. Body functions comprised pain at rest and following grip- and pinch-strength testing, and was self-reported on an NRS (scale: 0-10, 0 = no pain, 10 = severe pain). Activity and participation levels were self-reported with the Measure of Activity Performance of the Hand (MAP)-Hand questionnaire (1-4; 1 = no activity problems, 4 = severe activity problems)^30^ and the Disabilities of the Arm, Shoulder, and Hand questionnaire (QuickDASH; 0-100; 0 = no disability, 100 = severe disability)^31^ (Table 1).

**Table 1.** Baseline Demographic and Clinical Characteristics of 180 Patients Referred to Surgical Consultation Due to Carpometacarpal Osteoarthritis.

**Personal factors**	
**Age, years, mean (SD)**	63 (7.6)
**Women, n (%)**	142 (79)
**Living alone, n (%)**	35 (19)
**Employed, n (%)**	91 (51)
**Motivation for CMCJ surgery (NRS 0-10, 0 = no motivation), median (IQR)**	5 (3-8)
**Body structures**	
**Radiographic CMCJ OA severity (KLG grade 0-4, 0 = no CMCJ OA), median (IQR)**	3 (3-4)
**Body functions**	
**Pain following measure of pinch strength test (NRS 0-10, 0 = no pain), median (IQR)**	4 (2-6)
**Pain following measure of grip strength test (NRS 0-10, 0 = no pain), median (IQR)**	3 (2-5)
**Pain at rest (NRS 0-10, 0 = no pain), median (IQR)**	3 (1-4)
**Activity and participation**	
**Hand activity performance (MAP-Hand 1-4, 1 = no activity problems), mean (SD)**	1.97 (0.42)
**Disabilities of the arm, shoulder and hand (QuickDASH 0-100, 0 = no disability), mean (SD)**	36.88 (16.52)

*Note*. Numbers are reported as mean and standard deviation (*SD*), number and proportions (%), or median and interquartile range (IQR). Radiographic carpometacarpal joint osteoarthritis (CMCJ OA) severity is classified using modified Kellgren-Lawrence grade (KLG) scale (grade 0-4, 2 ≥ CMCJ OA). Pain is measured using Numeric Rating Scale (NRS: 0-10, 0 = no pain). Grip and pinch strength is measured in Newton (N) using the Grippit electronic instrument. Hand activity performance is measured using Measure of Activity Performance of the Hand (MAP-Hand) (score: 1-4, 1 = no activity problems). Disabilities of the arm, shoulder and hand is measured using Disabilities of the Arm, Shoulder, and Hand questionnaire (QuickDASH) (score: 0-100, 0 = no disability).

Data were collected by 6 experienced occupational therapists (RN, SD, REME, ÅK, KHM, MO) at the 3 hospitals.

### Analyses

Patient goals were linked to domains in the ICF coding system^29^ based on the linking rules of Cieza et al.^32^ First, the actual meaning of each goal was identified. Then, the goal was linked to the most relevant ICF code at the third level (3 digits). For goals that were not sufficiently specified to be linked or were not covered by an ICF code, we used the closest “unspecified” code option. Additionally, the response of patient might contain several goals, and thus, they would be linked to several ICF codes. For example, the response: “*To become pain-free and to improve my ability to use my hand in daily activities” was linked to ICF code b280 (sensation of pain), d445 (hand and arm use)*, and d230 (carrying out daily routines).

Two researchers (EMHG, ATT) that were familiar with the ICF linking rules linked approximately 20% of the all the patient responses independently. The codes were then compared, and when the 2 researchers had selected different codes, a third researcher (IK) was consulted for the final decision on the most appropriate code. After a consensus had been reached, 1 researcher (EMHG) linked the remaining answers.

Based on their goals, each patient was allocated to 1 of 3 groups. To explore differences in baseline self-reported pain and function between these groups, we used the One-Way between-groups analysis of variance (ANOVA) test for normally distributed variables. Results are reported as the mean and standard deviation (SD). We used the Kruskal-Wallis test for variables with a skewed distribution. Those results are reported as median and interquartile range (IQR).

The main indication for CMCJ surgery is persistent pain and reduced function.^5,11,12^ Pain and function measures and personal factors were therefore used as covariates to investigate factors that characterized patients that were highly motivated for surgery. As no cut-off value for the motivation question (measured on NRS) exists, we used the 75th percentile from this sample as a cut-off to identify highly motivated patients (individual NRS score ≥ 8 indicated highly motivated patients). Crude and adjusted logistic regression analyses were performed to compare patients highly motivated for surgery to those with less motivation. Correlation analyses were performed to investigate covariate multicollinearity. Pain following the pinch-strength test was highly correlated with pain following the grip-strength test, and the Disabilities of the Arm, Shoulder, and Hand questionnaire (QuickDASH) result was highly correlated with the MAP-Hand result. Therefore, we removed pain following the pinch-strength test and the QuickDASH results from further regression analyses. Variables from the crude analyses were included in the adjusted logistic regression model when their bivariate association with the motivation variable was *P* < 0.25. A backward removal regression analysis was performed to exclude variables that showed p > 0.05 in the adjusted analysis. Age and gender were forced into the model, regardless of p-value. Results of the regression analyses are expressed as odds ratio (OR) and 95% confidence interval (95% confidence interval [CI]). The amount of variation explained by the model was evaluated with the Nagelkerke *R*^2^ calculation.

## Results

A total of 180 patients were included in the study. The mean age was 63 years (SD = 7.6). Most patients were women (79%), 1 in 5 lived alone, and half of the sample was employed (Table 1).

We identified 261 different goals for CMCJ surgery that were linked to 15 different ICF codes (Table 2). Of these, 143 (55%) goals were linked to the body function domain (b) and 115 (44%) goals were linked to the activity and participation domain (d). One goal was linked to the body structure domain (s), and 2 goals were linked to the environmental factor domain (e). The goals were predominantly linked to 2 domains: (1) the body function domain (b280), which described (reductions in) sensation of pain (130 goals), and (2) the activity and participation domain (d445), which described (improved) arm and hand use (74 goals). In addition, patients frequently reported goals linked to carrying out daily routines (d230), acquiring, keeping, and terminating a job (d845), and muscle power function (b730). One goal could not be linked to an ICF code: “*Prevent further development of the ailments.*” Therefore, this goal was excluded from further analyses.

**Table 2.** Goals for Surgery Described by 180 Patients Referred for Surgical Consultation Due to Carpometacarpal Osteoarthritis, and Linked to Domains and Codes in the ICF.

ICF domain	ICF code	Description	n
**Body function**			
	b280	Sensation of pain	130
	b710	Mobility of joint function	1
	b729	Function of the joints and bones	2
	b730	Muscle power function	10
**Body structure**			
	s730	Structure of upper extremity	1
**Activity and participation**			
	d230	Carrying out daily routine	15
	d440	Fine hand use	4
	d445	Arm and hand use	74
	d570	Looking after one’s health	3
	d599	Self-care, unspecified	1
	d699	Major life areas, unspecified	1
	d845	Acquiring, keeping or terminating a job	12
	d920	Recreation and leisure	5
**Environmental factors**			
	e120	Products and technology for personal indoor and outdoor mobility and transportation	1
	e355	Health professionals	1

*Note.* ICF = International Classification of Function, Disability and Health.

Based on their goals (Table 2), each patient was allocated to 1 of 3 groups: Group 1: reducing pain (n = 73); Group 2: improving function, activity, and participation (n = 57); and Group 3: both reducing pain and improving function, activity, and participation (n = 48). One patient reported a goal that did not fit within 1 of the 3 groups (“*To get a straight hand”*); therefore, that patient was excluded from this analysis.

Patients in Group 1 reported less activity limitations and participation restrictions (reflected as lower scores on the MAP-Hand and QuickDASH questionnaires) compared to patients in the other 2 groups. However, we found no statistically significant between-group differences in any of the measures (Table 3).

**Table 3.** Difference in Pain and Functional Measures Between Groups According to Goals for Carpometacarpal Osteoarthritis (CMCJ OA) Surgery in 180 Patients Referred to Surgical Consultation Due to CMCJ OA.

Pain and Functional Measures	Group 1: Pain motivated patients (n = 73, 41%)	Group 2: Function, activity and participation motivated patients (n = 57, 32%)	Group 3: Pain, function, activity and participation motivated patients (n = 48, 27%)	*P* value
Pain following pinch strength test (NRS: 0-10, 0 = no pain), median (IQR)	3.3 (2.0-5.9)	4.0 (2.0-5.5)	4.0 (1.5-5.0)	.70
Pain following grip strength test (NRS: 0-10, 0 = no pain), median (IQR)	3.0 (2.0-5.0)	4.0 (2.0-4.8)	2.5 (0.9-5.0)	.25
Pain at rest (NRS: 0-10, 0 = no pain), median (IQR)	3.0 (1.3-4.0)	3.0 (1.0-4.0)	2.0 (1.0-5.0)	.60
MAP-Hand (1-4, 1 = no activity problems), mean (SD)	1.89 (0.41)	2.02 (0.41)	2.05 (0.44)	.08
QuickDASH (0-100, 0 = no disability), mean (*SD*)	34.05 (16.91)	38.03 (14.09)	40.24 (17.36)	.11

*Note*. Pain is self-reported using Numeric Rating Scale (NRS score: 0-10, 0 = no pain). CMCJ OA = carpometacarpal joint osteoarthritis; IQR = inter quartile range; MAP-Hand = measure for activity performance of the hand (score: 1-4, 1 = no activity problems); QuickDASH = Disabilities of the Arm, Shoulder and Hand questionnaire (score: 0-100, 0 = no disability).

Fifty-six (31%) patients were classified as highly motivated for CMCJ surgery. The adjusted logistic regression analyses (Table 4) showed that the odds of being highly motivated for surgery was 4-fold higher for patients with more activity limitations (OR = 4.00, 95% CI = 1.67-9.58), and more than 3-fold higher for those living alone (OR = 3.18, 95% CI = 1.38-7.36). Furthermore, the OR for age was 0.94 (95% CI = 0.90-0.98), indicating that younger patients were more likely to be highly motivated for surgery. This model explained 15.3% of the variation in motivation for CMCJ surgery.

**Table 4.** Logistic Regression Analyses in a Sample of 180 Patients With Carpometacarpal Osteoarthritis (CMCJ OA) Predicting Likelihood for Being Highly Motivated for CMCJ Surgery; Crude and Adjusted Analyses.

		Bivariate crude analyses		Adjusted analysis	
Covariates		OR (95% CI)	*P* value	OR (95% CI)	*P* value
Age (in years)		0.96 (0.91-1.00)	.039*	0.94 (0.90-0.98)	.008
Gender	Male	1		1	
	Female	0.98 (0.46-2.13)	.97*	0.50 (0.20-1.21)	.12
Living alone	No	1		1	
	Yes	2.19 (1.03-4.67)	.043*	3.18 (1.38-7.36)	.007
Radiographic CMCJ OA	No	1			
	Yes	0.43 (0.03-7.06)	.56		
Pain following grip strength test (0-10, 0 = no pain)		1.16 (1.01-1.33)	.036*		
Pain at rest (0-10, 0 = no pain)		1.11 (0.97-1.28)	.14*		
MAP-Hand (1-4, 1 = no activity problems)		2.85 (1.30-6.22)	.009*	4.00 (1.67-9.58)	.002

*Note*. Radiographic CMCJ OA severity is classified using modified Kellgren-Lawrence grade scale (Grades 0-4: 0 = no CMCJ OA). Pain is measured using Numeric Rating Scale (NRS score: 0-10, 0 = no pain). Hand activity performance is measured with mean score of MAP-Hand (score: 1-4, 1 = no activity problems). OR = odds ratio; CI = confidence interval; MAP-Hand = measure for activity performance of the hand (score 1-4, 1 = no activity problems).

* Variables included in the backward removal regression analysis (*P* < .5).

## Discussion

This study is one of very few studies to explore patient goals and motivation for CMCJ surgery. The results showed that, among patients referred to surgical consultations for CMCJ OA, the most frequently reported goals for undergoing CMCJ surgery were reduced pain and improved hand and arm use. Differences in patient goals were not reflected in differences in self-reports of pain and function. High motivation for surgery was significantly associated with more activity limitations, living alone, and younger age.

The finding that reduced pain and improved hand and arm use were the most frequently reported goals for undergoing surgery are consistent with findings in previous studies,^6,21^ and supports the recommendation that persistent pain and reduced function, caused by the affected OA joint, can help surgeons in the decision on whether or not to conduct surgery.^5,11,12^ Nevertheless, we did not find any statistical significant differences between the 3 groups with different goals; they all reported approximately the same levels of pain and activity limitations.

Treatment choices should be based on a common understanding of the treatment goals, benefits, and risks.^19,20^ A patient-specific instrument that captures patients’ individual goals related to functional limitations can be used as a supplement to the standardized measures of pain and activity limitations. This tool might provide additional valuable information for the decision-making process.^33,34^

In our study, more self-reported activity limitations quadrupled the odds of high motivation for surgery. Although surgery represents a treatment alternative for patients with the most severe disease, all patients should first receive the core treatment,^5^ because this has been found to have positive effects on pain and activity performance.^35,36^ Our previous analyses showed that only 20% of patients in the RCT had received nonpharmacological treatments prior to the surgical consultation.^27^ Therefore, patients and GPs should be educated in evidence-based treatment recommendations^5^ to ensure that those with symptomatic hand OA receive the recommended core treatment before surgery is considered.

Living alone was also strongly associated with high motivation for surgery. In a previous study, patients living with a partner stated that their partner’s support in managing objects, self-care, and household activities was important in performing everyday activities. In contrast, living alone provided less opportunity to allocate challenging everyday-tasks to others; thus, living alone was found to add extra disease burden.^37^ That same study also showed that few patients had received assistive devices, and very few had knowledge about the availability of assistive devices for alleviating activity limitations. The use of assistive devices and activity adaptations are frequently reported as self-management strategies,^38^ and they have been found to improve activity performance significantly in patients with hand OA.^36^ Therefore, particular attention should be given to patients with CMCJ OA that live alone and those with sparse support networks^37^ when considering interventions that could contribute to improving activity performance and increasing self-management.

In our study, younger patients were more likely to be highly motivated for CMCJ surgery than older patients. One explanation for this finding might be that younger patients live an active life with high demands regarding participation in several family and social arenas, such as work. In a study that included more than 57 000 participants with hand OA from 5 European countries, one third of those working reported work-related challenges.^39^ In addition, a qualitative study showed that younger patients worried about the financial implications of quitting work or retiring at an early age due to hand OA.^40^ Furthermore, 50% of patients in the current study were employed, and several reported that acquiring or keeping a job was their goal for surgery. Therefore, the disease impact on younger patients should be an area of future investigations, with a particular focus on work participation.

A strength of our study was the large study sample, which we recruited from 3 hospitals in different geographical areas. Furthermore, there were no missing responses with regard to the goals and motivation for surgery. One potential limitation was the suggested response alternatives for patients with no clear goals for surgery, which may have led to less variation in the goal descriptions. Additionally, because our results were based on cross-sectional data, we could not make any causal inferences regarding the associations between motivation for surgery and covariates. Furthermore, it may be that patients with more severe disease may likely report higher motivation for surgery, and thereby skew the data in that direction. However, our results show that neither radiographic severity nor pain predicted likelihood for being highly motivated for surgery. The initial open-ended question may have led patients to think about surgery as the first treatment choice. In retrospective, an alternative way of asking could be to encourage patients to explore motivation for (any) treatment, and then add a follow-up question on motivation for surgery.

In summary, reduced pain and improved arm and hand use were the most frequently reported goals for surgery. However, these goals were not reflected in self-reports of pain and function. High motivation for surgery was strongly associated with activity limitations, living alone, and younger age. Decisions on CMCJ OA surgery should be based on assessment and discussion of patients’ life situation, hand pain, activity limitations and goals, and motivation for surgery. According to the european league against rheumatism (EULAR) recommendations, previously received conservative and pharmacological treatment should also be evaluated.

## References

[1] Bijsterbosch J Visser W Kroon HM , et al. Thumb base involvement in symptomatic hand osteoarthritis is associated with more pain and functional disability. Ann Rheum Dis. 2010;69:585-587. 20124359 10.1136/ard.2009.104562

[2] Bukhave EB La Cour K Huniche L. The meaning of activity and participation in everyday life when living with hand osteoarthritis. Scand J Occup Ther. 2014;21:24-30. 24289728 10.3109/11038128.2013.857428

[3] Haugen IK Englund M Aliabadi P , et al. Prevalence, incidence and progression of hand osteoarthritis in the general population: the Framingham Osteoarthritis Study. Ann Rheum Dis. 2011;70:1581-1586. 21622766 10.1136/ard.2011.150078 PMC3867970

[4] Moriatis Wolf J Turkiewicz A Atroshi I , et al. Prevalence of doctor-diagnosed thumb carpometacarpal joint osteoarthritis: an analysis of Swedish health care. Arthritis Care Res (Hoboken). 2014;66:961-965. 24339432 10.1002/acr.22250

[5] Kloppenburg M Kroon FP Blanco FJ , et al. 2018 update of the EULAR recommendations for the management of hand osteoarthritis. Ann Rheum Dis. 2019;78:16-24. 30154087 10.1136/annrheumdis-2018-213826

[6] Tsehaie J Porsius JT Rizopoulos D , et al. Response to conservative treatment for thumb carpometacarpal osteoarthritis is associated with conversion to surgery: a prospective cohort study. Phys Ther. 2019;99:570-576. 30715532 10.1093/ptj/pzz009 PMC6489165

[7] Ahern M Skyllas J Wajon A , et al. The effectiveness of physical therapies for patients with base of thumb osteoarthritis: systematic review and meta-analysis. Musculoskelet Sci Pract. 2018;35:46-54. 29510316 10.1016/j.msksp.2018.02.005

[8] Brosseau L Thevenot O MacKiddie O , et al. The Ottawa Panel guidelines on programmes involving therapeutic exercise for the management of hand osteoarthritis. Clin Rehabil. 2018;32:1449-1471. 29911409 10.1177/0269215518780973

[9] Kloppenburg M van Beest S Kroon FPB . Thumb base osteoarthritis: a hand osteoarthritis subset requiring a distinct approach. Best Pract Res Clin Rheumatol. 2017;31:649-660. 30509411 10.1016/j.berh.2018.08.007

[10] Conaghan PG Cook AD Hamilton JA , et al. Therapeutic options for targeting inflammatory osteoarthritis pain. Nat Rev Rheumatol. 2019;15:355-363. 31068673 10.1038/s41584-019-0221-y

[11] Higgenbotham C Boyd A Busch M , et al. Optimal management of thumb basal joint arthritis: challenges and solutions. Orthop Res Rev. 2017;9:93-99. 30774481 10.2147/ORR.S138809 PMC6209361

[12] Wajon A Vinycomb T Carr E , et al. Surgery for thumb (trapeziometacarpal joint) osteoarthritis. Cochrane Database Syst Rev. 2015;2:CD004631. 10.1002/14651858.CD004631.pub4 PMC6464627 25702783

[13] Donato D Abunimer AM Abou-Al-Shaar H , et al. First carpometacarpal joint denervation for primary osteoarthritis: technique and outcomes. World Neurosurg. 2019;122:e1374-e1380. 10.1016/j.wneu.2018.11.061 30465956

[14] De Maio F Farsetti P Potenza V , et al. Surgical treatment of primary trapezio-metacarpal osteoarthritis by trapeziectomy and ligament reconstruction without tendon interposition. Long-term results of 50 cases. J Orthop Traumatol. 2019;20:25. 31267254 10.1186/s10195-019-0532-4 PMC6606683

[15] Vermeulen GM Slijper H Feitz R , et al. Surgical management of primary thumb carpometacarpal osteoarthritis: a systematic review. J Hand Surg Am. 2011;36:157-169. 21193136 10.1016/j.jhsa.2010.10.028

[16] Wolf JM Atroshi I Zhou C , et al. Sick leave after surgery for thumb carpometacarpal osteoarthritis: a population-based study. J Hand Surg Am. 2018;43:439-447. 29428245 10.1016/j.jhsa.2017.11.019

[17] Wouters RM Tsehaie J Hovius SER , et al. Postoperative rehabilitation following thumb base surgery: a systematic review of the literature. Arch Phys Med Rehabil. 2018;99:1177-1212.e2. 10.1016/j.apmr.2017.09.114 29030095

[18] Deutch Z Niedermeier SR Awan HM. Surgeon preference, influence, and treatment of thumb carpometacarpal arthritis. Hand (N Y). 2018;13:403-411. 28685589 10.1177/1558944717717506 PMC6081787

[19] Stiggelbout AM Pieterse AH De Haes JC. Shared decision making: concepts, evidence, and practice. Patient Educ Couns. 2015;98:1172-1179. 26215573 10.1016/j.pec.2015.06.022

[20] Boss EF Mehta N Nagarajan N , et al. Shared decision making and choice for elective surgical care: a systematic review. Otolaryngol Head Neck Surg. 2016;154:405-420. 26645531 10.1177/0194599815620558 PMC4857133

[21] Wilkens SC Menendez ME Ring D , et al. QuickDASH score is associated with treatment choice in patients with trapeziometacarpal arthrosis. Hand (N Y). 2017;12:461-466. 28832210 10.1177/1558944716677937 PMC5684932

[22] Cronstrom A Nero H Dahlberg LE. Factors associated with patients’ willingness to consider joint surgery after completion of a digital osteoarthritis treatment program: a prospective cohort study. Arthritis Care Res (Hoboken). 2019;71:1194-1201. 30298990 10.1002/acr.23772 PMC6771662

[23] Selten EMH Geenen R Schers HJ , et al. Treatment beliefs underlying intended treatment choices in knee and hip osteoarthritis. Int J Behav Med. 2018;25:198-206. 28664420 10.1007/s12529-017-9671-2

[24] Traumer L Sorensen EE Kusk KH , et al. Investigating the motives of patients with knee OA undergoing a TKR: a qualitative interview study. Musculoskeletal Care. 2018;16:380-387. 29656439 10.1002/msc.1244

[25] Kjeken I Eide REM Klokkereide A , et al. Does occupational therapy reduce the need for surgery in carpometacarpal osteoarthritis? Protocol for a randomized controlled trial. BMC Musculoscelet Disord. 2016;17:473. 10.1186/s12891-016-1321-3 PMC5109819 27842579

[26] Gravas EMH Osteras N Nossum R , et al. Does occupational therapy delay or reduce the proportion of patients that receives thumb carpometacarpal joint surgery? A multicentre randomised controlled trial. RMD Open. 2019;5:e001046. 10.1136/rmdopen-2019-001046 PMC6861078 31798953

[27] Gravas EMH Tveter AT Nossum R , et al. Non—pharmacological treatment gap preceding surgical consultation in thumb carpometacarpal osteoarthritis—a cross-sectional study. BMC Musculoskelet Disord. 2019;20:180. 31039774 10.1186/s12891-019-2567-3 PMC6492412

[28] Villafane JH Valdes K. Combined thumb abduction and index finger extension strength: a comparison of older adults with and without thumb carpometacarpal osteoarthritis. J Manipulative Physiol Ther. 2013;36:238-244. 23719517 10.1016/j.jmpt.2013.05.004

[29] World Health Organization. International Classification of Functioning, Disability and Health (ICF). World Health Organization; 2018. http://www.who.int/classification/icf/en/.

[30] Fernandes L Grotle M Darre S , et al. Validity and responsiveness of the Measure of Activity Performance of the Hand (MAP-Hand) in patients with hand osteoarthritis. J Rehabil Med. 2012;44:869-876. 22948123 10.2340/16501977-1035

[31] Hammond A Prior Y Tyson S. Linguistic validation, validity and reliability of the British English versions of the Disabilities of the Arm, Shoulder and Hand (DASH) questionnaire and QuickDASH in people with rheumatoid arthritis. BMC Musculoskelet Disord. 2018;19:118. 29661183 10.1186/s12891-018-2032-8 PMC5902839

[32] Cieza A Fayed N Bickenbach J , et al. Refinements of the ICF Linking Rules to strengthen their potential for establishing comparability of health information. Disabil Rehabil. 2019;41:574-583. 26984720 10.3109/09638288.2016.1145258

[33] Porter I Goncalves-Bradley D Ricci-Cabello I , et al. Framework and guidance for implementing patient-reported outcomes in clinical practice: evidence, challenges and opportunities. J Comp Eff Res. 2016;5:507-519. 27427277 10.2217/cer-2015-0014

[34] Lavallee DC Chenok KE Love RM , et al. Incorporating patient-reported outcomes into health care to engage patients and enhance care. Health Aff (Millwood). 2016;35:575-582. 27044954 10.1377/hlthaff.2015.1362

[35] Tveter AT Nossum R Eide REM , et al. Op0161 HPR short—term effect of occupational therapy intervention on hand function and pain in patients with thumb base osteoarthritis—secondary analyses of a randomized controlled trial. Ann Rheum Dis. 2019;78:155-156.30282668

[36] Kjeken I Darre S Smedslund G , et al. Effect of assistive technology in hand osteoarthritis: a randomised controlled trial. Ann Rheum Dis. 2011;70:1447-1452. 21571733 10.1136/ard.2010.148668

[37] Bukhave EB Huniche L . Activity problems in everyday life—patients’ perspectives of hand osteoarthritis: “try imagining what it would be like having no hands.” Disabil Rehabil. 2014;36:1636-1643. 24308906 10.3109/09638288.2013.863390

[38] Kjeken I Darre S Slatkowsky-Cristensen B , et al. Self-management strategies to support performance of daily activities in hand osteoarthritis. Scand J Occup Ther. 2013;20:29-36. 22376127 10.3109/11038128.2012.661457

[39] Kingsbury SR Gross HJ Isherwood G , et al. Osteoarthritis in Europe: impact on health status, work productivity and use of pharmacotherapies in five European countries. Rheumatology (Oxford). 2014;53:937-947. 24489012 10.1093/rheumatology/ket463

[40] Hill S Dziedzic KS Ong BN. The functional and psychological impact of hand osteoarthritis. Chronic Illn. 2010;6:101-110. 20444766 10.1177/1742395309345614

